# Measurement of Thermal Stress by X-ray Nano-Diffraction in (111)-Oriented Nanotwinned Cu Bumps for Cu/SiO_2_ Hybrid Joints

**DOI:** 10.3390/nano13172448

**Published:** 2023-08-29

**Authors:** Wei-You Hsu, Shih-Chi Yang, You-Yi Lin, Wan-Zhen Hsieh, King-Ning Tu, Wei-Lan Chiu, Hsiang-Hung Chang, Ching-Yu Chiang, Chih Chen

**Affiliations:** 1 Department of Materials Science and Engineering, National Yang Ming Chiao Tung University, Hsinchu 30010, Taiwan; mice803.c@nycu.edu.tw (W.-Y.H.); nanotwinnedcu.en10@nycu.edu.tw (S.-C.Y.); yylin0106.en10@nycu.edu.tw (Y.-Y.L.); 2 National Synchrotron Radiation Research Center, Hsinchu 30076, Taiwan; hsieh.wz@nsrrc.org.tw; 3 Department of Materials Science and Engineering, City University of Hong Kong, Hong Kong; kntu@cityu.edu.hk; 4 Department of Electrical Engineering, City University of Hong Kong, Hong Kong; 5 Electronics and Optoelectronics System Research Laboratories, Industrial Technology Research Institute (ITRI), Hsinchu 30010, Taiwan; arthurchiu@itri.org.tw (W.-L.C.); mikechang@itri.org.tw (H.-H.C.)

**Keywords:** synchrotron radiation, X-ray nanodiffraction, thermal strain distribution, Cu/SiO_2_ hybrid bonding, nanotwinned Cu

## Abstract

X-ray nanodiffraction was used to measure the thermal stress of 10 µm nanotwinned Cu bumps in Cu/SiO_2_ hybrid structures at −55 °C, 27 °C, 100 °C, 150 °C, and 200 °C. Bonding can be achieved without externally applied compression. The X-ray beam size is about 100 nm in diameter. The Cu bump is dominated by (111) oriented nano-twins. Before the hybrid bonding, the thermal stress in Cu bumps is compressive and remains compressive after bonding. The average stress in the bonded Cu joint at 200 °C is as large as −169.1 MPa. In addition, using the strain data measured at various temperatures, one can calculate the effective thermal expansion coefficient (CTE) for the 10 µm Cu bumps confined by the SiO_2_ dielectrics. This study reports a useful approach on measuring the strain and stress in oriented metal bumps confined by SiO_2_ dielectrics. The results also provide a deeper understanding on the mechanism of hybrid bonding without externally applied compression.

## 1. Introduction

Owing to the demand for high-performing computing (HPC) devices and high bandwidth memory (HBM), the density of micro-bumps in 3D IC technology has increased significantly. Moreover, the solder joint has destructive reliability issues with decreasing pitch, such as sidewall wetting, brittle intermetallic compound (IMC) formation, and bridge failure [1,2,3]. Thus, Cu/SiO_2_ or Cu/SiCN hybrid bonds have replaced the solder joints in HPC devices [4,5,6,7,8,9,10,11,12,13,14]. However, the current temperature to achieve Cu hybrid bonding is about 300 °C. Several studies propose different approaches to achieve low-temperature bonding, including (111)-oriented nano-twinned Cu, adoption of the passivation layer, and plasma treatment [10,15,16,17,18,19,20,21,22,23,24]. Using the rapid surface diffusion on (111)-preferred surfaces, one can reduce the bonding temperature to 150 °C [16].

Nanotwinned Cu (NT-Cu) has high strength and low resistivity compared with nanocrystalline Cu and coarse-grained Cu [25]. The common methods of fabrication of NT-Cu are sputter and pulse electroplating, but the cost of sputter and the time duration of pulse electroplating is not suitable for the micro-electrical industry [25,26,27,28,29,30,31]. In 2012, Chen’s group introduced NT-Cu fabricated by direct-current electroplating [32]. In particular, the highly (111)-oriented NT-Cu has the highest surface diffusivity and a low oxidation rate [33,34,35], so it is highly suitable for Cu direct bonding. Also, the NT-Cu is thermal stable up to 300 °C and has high resistance against electromigration and high strength [36,37,38,39]. The NT-Cu has abnormal grain growth, which can enhance the bonding strength [40], so (111)-oriented NT-Cu would be a promising candidate of Cu hybrid bonding.

**Figure 1:**
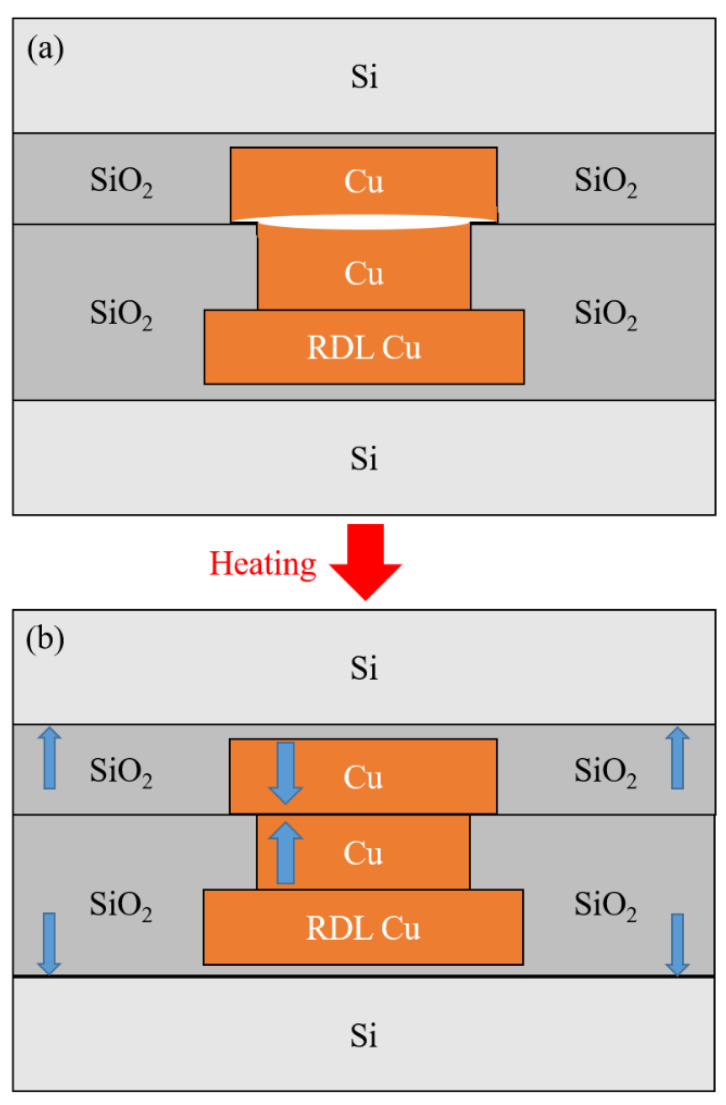
Structure of Cu/SiO_2_ hybrid joints. (**a**) Schematic drawing after the SiO_2_-SiO_2_ bonding near room temperature. (**b**) Schematic structure showing the Cu expanding die to CTE mismatch at elevated temperature.

The kinetic mechanism for forming the Cu/SiO_2_ joints is surface creep, illustrated in Figure 1 [16,19]. Those Cu bumps with a slight recess in SiO_2_ vias are aligned and pressurized at near room temperature, and the SiO_2_–SiO_2_ dielectric first bonded to each other. After the pressurization process, the Si wafer pair was heated to a high temperature of 150–300 °C [41,42]. Because the thermal expansion coefficient (CTE) of Cu is larger than that of SiO_2_, the two Cu bumps expand to touch each other and provide the compressive stress gradient needed for creep to occur, as depicted in Figure 1a,b. It is worth noting that it does not need any external compression during the heating process. The value of the generated stress due to the CTE mismatch was simulated by finite element analysis [43,44,45]. However, there is no experimental measurement of the strain/stress value in the Cu joints near the bonding temperature so far.

In this study, the strain in the 8 µm Cu pad, 10 µm Cu pad, and 8 µm Cu joint was in situ measured by synchrotron X-ray at −55, 27, 100, 150, and 200 °C using nanobeam diffraction. The resolution of the beam size is 100 nm. Therefore, we can obtain the strain and stress distribution in the Cu joints at different temperatures, which can provide a deeper understanding than the previous studies on the fabrication and reliability of Cu–Cu hybrid joints.

## 2. Materials and Methods

**Figure 2:**
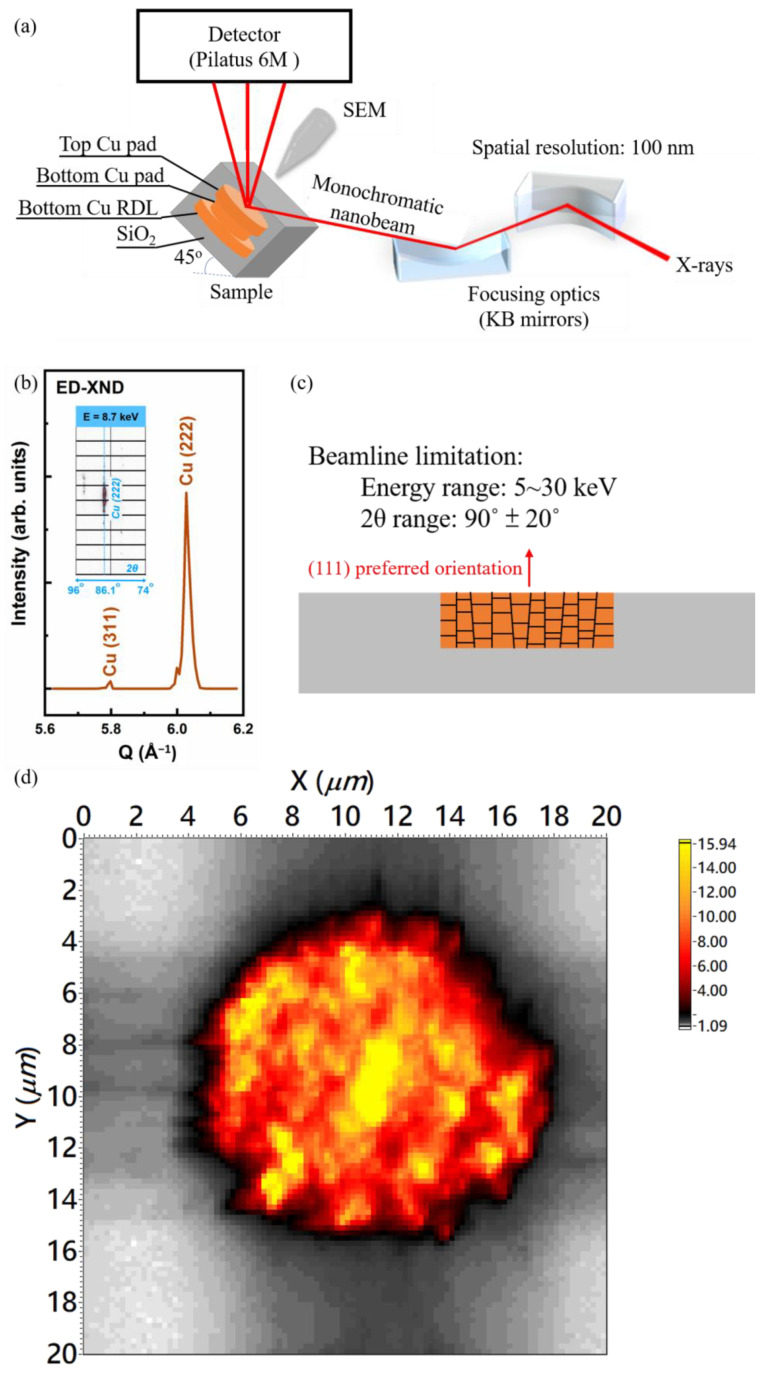
(**a**) Schematic diagram of measurement at BL21A. (**b**) The measured method of ED-XND. (**c**) The schematic diagram of the (111)-oriented Cu in SiO_2_ via and beamline limitation. (**d**) The spatial distribution of diffraction peak intensity from the Cu (222) crystal plane.

This study used three types of samples to measure the thermal strain: the top die, bottom die, and bonded joint. The top die (6 × 6 mm^2^) consists of arrays of Cu bumps with 10 µm diameter, and the bottom die (15 × 15 mm^2^) comprises arrays of Cu bumps with 8 µm diameter on top of Cu redistribution layers (RDLs). The thickness of Cu bumps and Cu RDLs is ~1.25 µm; thus, the total thickness of the top die, bottom die, and bonded joint is ~1.25, ~2.5, and ~3.75 µm, respectively. The fabrication method of the hybrid Cu/SiO_2_ joints was reported in a previous study [16]. Nano-twinned Cu with highly (111) preferred orientation was fabricated by electrodeposition [32]. We adopted a complete package without being cut or ground for the thermal strain measurement, so the strain/stress distribution is similar to the state during bonding. The thickness of the top Si wafer is only 100 µm, so the X-ray can penetrate the entire device and can directly detect the diffraction signals from Cu bumps. The strain distribution was performed by using the X-ray nano-diffraction (XND) beamline, BL21A station, at Taiwan Photon Source (TPS) at the National Synchrotron Radiation Research Center (NSRRC) in Hsinchu, Taiwan. The instrument accommodates an FE-SEM and X-ray fluorescence (nano-XRF) system to navigate the 100 nm focused white/mono X-rays on a specific bump region. The energy range of the focused X-ray covers from 5 to 30 keV, and for the experiment, we quickly switched to the mono X-ray beam by introducing the 4-bunch crystal monochromator (4BCM) [46]. The entire system was combined in a high-vacuum chamber (10^−7^ Torr) to prevent air scattering of diffracted signals, as shown in Figure 2a.

In the nano-diffraction technique, to avoid the displacement caused by the rotation of the sample, we fixed the sample at an angle and scanned the energy of the incident X-ray instead of rotating the sample. Considering Bragg’s equation, scanning the incident X-ray energy allowed us to obtain the distribution of the lattice spacing of the sample at a fixed angle. This technique is called energy-dispersive nano-diffraction (ED-XND), as shown in Figure 2b. In this study, the sample was fixed at a 45° angle, and the diffraction signal was collected using a large-area detector (Pilatus 6M, Dectris, Baden, Switzerland) at a reflection geometry 90° above the sample stage. We chose a region of interest (2-theta ROI) on the detector and converted the incident X-ray energy into scattering wave vector, Q, or reciprocal lattice units, r.l.u.; an 1-D diffraction pattern from a nanometer scale region can be obtained for structural determination. We selected the X-ray energy from a lattice plane vector and scanned the sample for two-dimensional mapping; by analyzing the angular shift of this crystal plane on the detector, the crystal plane and strain distribution can be observed in real space.

The strain caused by the CTE mismatch is of interest at different temperatures, especially near the bonding temperatures; also, we were concerned about the reliability during thermal cycling test of hybrid bonding, so we measured the temperature at low temperature [15,16]. Therefore, in this study, the sample temperature was set at −55, 27, 100, 150, and 200 °C. The thermal strain at various temperatures was calculated by Equation (1).

(1) $$\varepsilon =\frac{∆d}{d_{0,T}}=\frac{d_{s}-d_{0,T}}{d_{0,T}}$$

where *ε* is the lattice strain, $d_{s}$ is the strain lattice parameter, and $d_{0,T}$ is the unstrained lattice parameter at temperature *T*, which would change with temperature, so the unstrained lattice parameter $d_{0,T}$ was calculated by Equation (2).

(2) $$d_{0,T}=d_{0,25{}^{\circ}C}\times (T-298)\times (1+\alpha )$$

where $d_{0,25{}^{\circ}C}$ is the unstrained lattice parameter at 25 °C, and *α* is the coefficient of thermal expansion. At room temperature (25 °C), the unstrained lattice parameter of Cu was taken as 0.3615 nm, which is the lattice constant in powder diffraction (PDF 00-004-0836), and the coefficient of thermal expansion was 16.99 × 10^−6^/°C at 25 °C. The microstructure of the top die, the bottom die, and the bonded joint was observed with a focused ion beam (FIB).

## 3. Results and Discussion

### 3.1. Diffraction Intensity and Microstructure

The thermal strain/stress generated by the mismatch of CTE provides the driving force for the Cu–Cu bonding. According to the ED-XND technique, we collected a diffraction pattern in the range of 1000 eV with a step of 10 eV. From the results, we observed a strong diffraction signal on the detector that was close to 86° two theta degrees when the incident mono X-ray energy was 8.7 keV. After calculation, this diffraction signal corresponds to Cu (222) and was oriented along the normal direction of the sample surface. Based on our previous studies, the surface (111) orientation of the nano-twin in the Cu bump on the top and bottom die is about 78% [16]. Hence, to understand the variation of strain between Cu–Cu bumps, measuring the lattice changes of the Cu {111} family along the bonding direction is the optimal choice.

**Figure 3:**
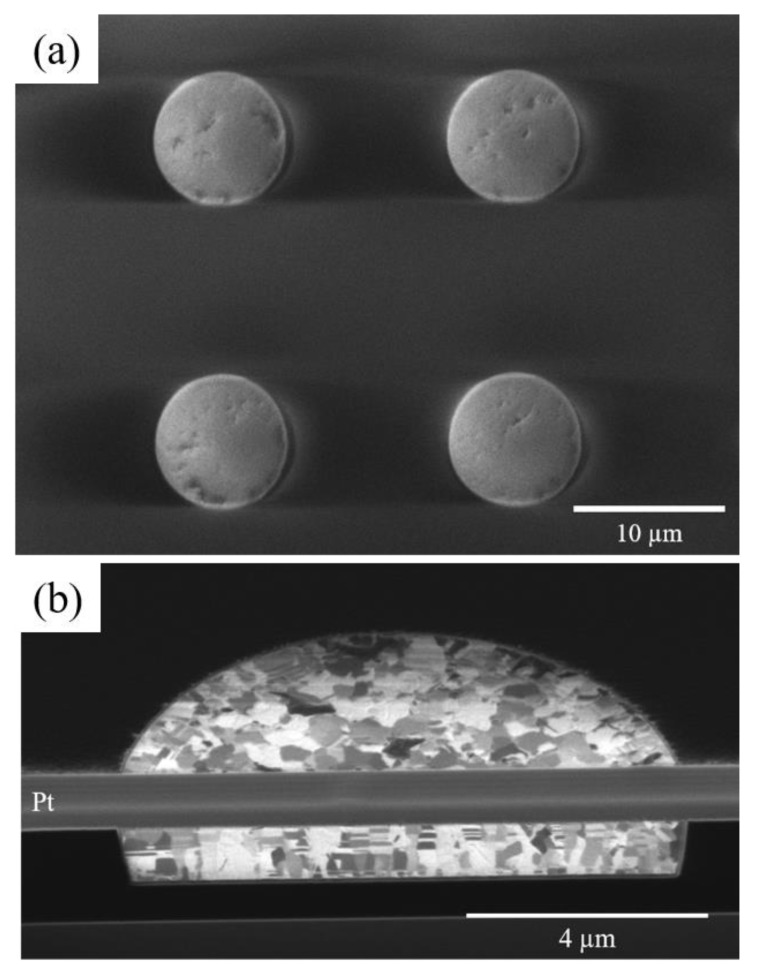

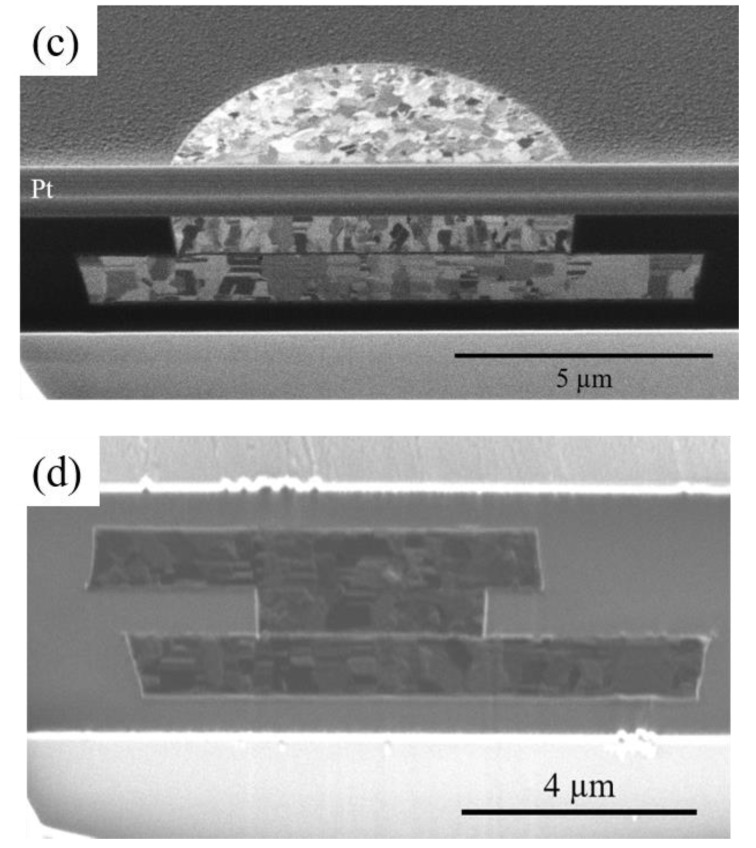
Microstructure of NT-Cu pad. (**a**) Plan-view SEM secondary electron image of top die NT-Cu pads. (**b**) Cross-sectional ion image of the top die NT-Cu pad. (**c**) Cross-sectional ion image of the bottom die NT-Cu pad. (**d**) Cross-sectional ion image of the bonded NT-Cu joint.

Due to the geometric limitations of the XND beamline and the surface orientation of the Cu bump, as shown in Figure 2c, we selected Cu (222) crystal plane, detected at 8.7 keV incident X-ray, to plot the distribution of a single Cu–Cu bump. Figure 2d shows the spatial distribution of diffraction peak intensity from the Cu (222) crystal plane. A precision stage (SmarAct) moved the sample at an interval of 200 nm for two-dimensional mapping, and the measured region is 20 µm × 20 µm, which is completely included in one Cu joint. The thickness of the top Si die was ground from 725 µm to 100 µm to enhance the diffraction signals from Cu. The secondary electron image and ion image of the top die and the bottom die are shown in Figure 3. Figure 3a is the secondary electron image of the top die. There are four Cu pads in Figure 3a. In this study, we only measured the thermal strain of one Cu pad or joint in every sample. Figure 3b–d show the cross-sectional FIB image of the top die Cu pad, the bottom die Cu pad, and the bonded Cu joint. As can be seen, the microstructure of all samples has a nano-twin structure. The thickness of the Cu bump and RDL is 1.25 µm in each Cu layer. Therefore, the diffractions were from all of the three Cu layers.

### 3.2. Thermal Strain/Stress Maps

**Figure 4:**
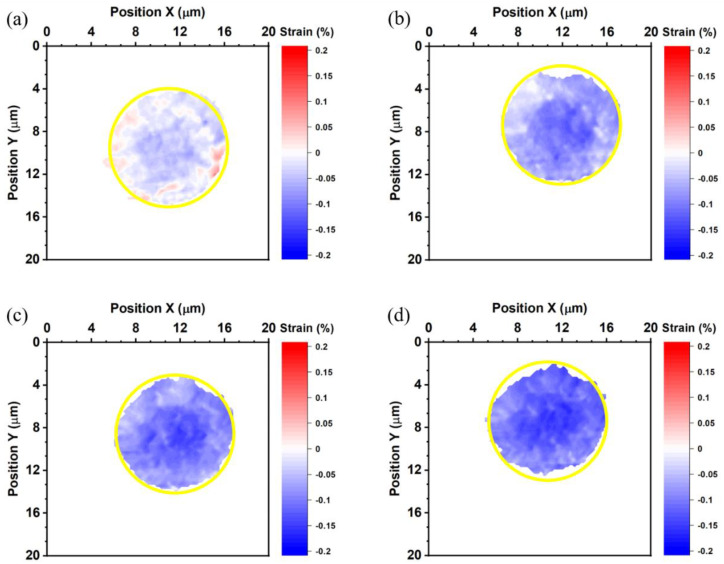
Thermal strain maps of the top die measured at (**a**) 27, (**b**) 100, (**c**) 150, and (**d**) 200 °C. The yellow circles represent the site of the bump.

The strains in the top die Cu pad, the bottom die Cu pad, and the bonded Cu joint were calculated by Equation (1). The average thermal strain of all specimen is listed in Table 1. Figure 4 shows the thermal strain maps of the top die measured at 27, 100, 150, and 200 °C. The positive value indicates tensile strain, and the negative value represents compressive strain. The yellow circles in Figure 4 locate the site of the bump.

At 27 °C, the top die Cu pad is under compressive strain, and compressive strain rises as the temperature increases. The thermal strain maps indicate higher compressive strain at the middle of the top die bump, as shown in Figure 4. We suspect that the non-uniform planarization in the Cu bumps caused the higher strain. It is reported that dishing may occur in the Cu via chemical-mechanical polishing (CMP). The Cu near the edge of the bump is thicker than that near the center of the bump [36]. Therefore, the Cu near the edge was compressed more during the bonding process, as illustrated in Figure 1a. The average thermal strain of the top die Cu pad is −0.018%, −0.07%, −0.089%, and −0.111%, at 27, 100, 150, and 200 °C, respectively.

**Figure 5:**
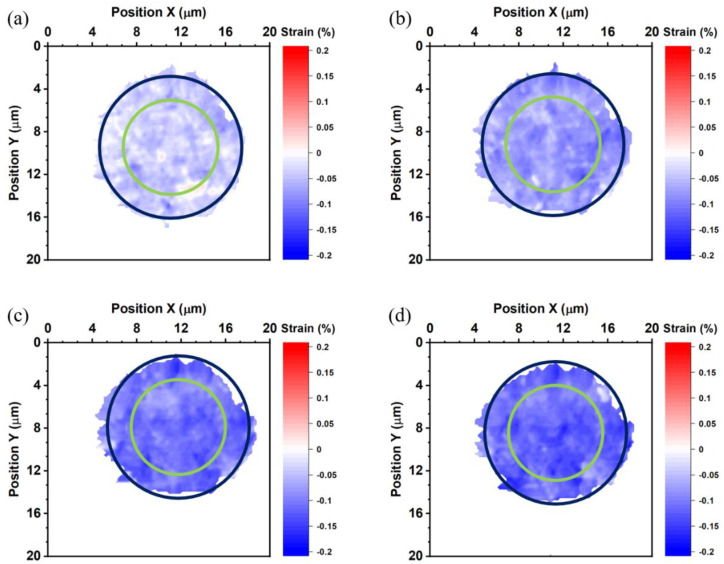
Thermal strain maps of the bottom die measured at (**a**) 27, (**b**) 100, (**c**) 150, and (**d**) 200 °C. The blue and green circles locate the site of the bump and the RDL, respectively.

**Figure 6:**
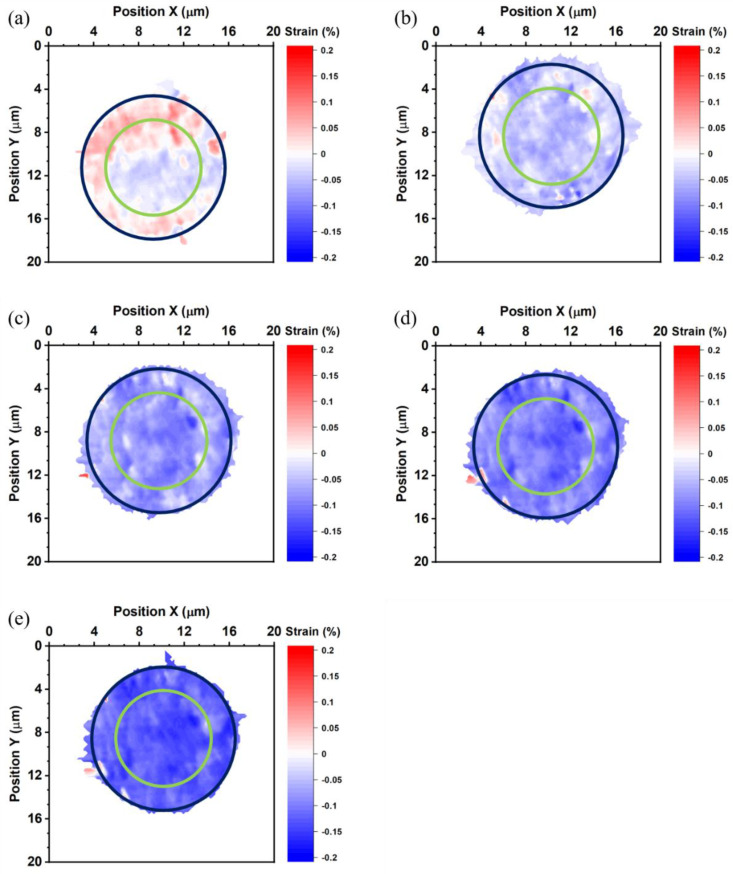
Thermal strain maps of the bonded joint measured at (**a**) −55, (**b**) 27, (**c**) 100, (**d**) 150, and (**e**) 200 °C. The blue and green circles represent the locations of the bump and the RDL, respectively.

Figure 5 shows the thermal strain maps of the bottom die Cu pad measured at 27, 100, 150, and 200 °C. The blue (large) and green (small) circles in Figure 5 locate the site of the bump and the RDL, respectively. The average thermal strain of the bottom die Cu pad is −0.037%, −0.074%, −0.105%, and −0.113% at 27, 100, 150, and 200 °C, respectively. Figure 6 shows the thermal strain maps of the bonded Cu joint measured at −55, 27, 100, 150, and 200 °C. In Figure 6, we used blue (large) and green (small) circles to locate the site of the bump and the RDL, respectively. The average thermal strain of the bonded Cu joint is 0.007%, −0.039%, −0.072%, −0.094%, and −0.121% at −55, 27, 100, 150, and 200 °C. However, the misalignment between bumps in the top and bottom dies could not be observed from these maps. The thermal strain maps of the bonded joint have higher strain at the edge of the bottom die RDL in Figure 6b–d. This is not observed in the thermal strain maps of the top die and the bottom die, so we suspect the higher strain was caused by the thermal-compression bonding process. The thermal-compression bonding may cause some defects at the edge of the bottom die RDL. The trend of the average thermal strain of all samples is the same. All samples obtain higher compressive strain with increasing temperatures.

Furthermore, the Young’s modulus of Cu was taken as 140 GPa to calculate the change of thermal strain to thermal stress with Equation (3) [37].

**Figure 7:**
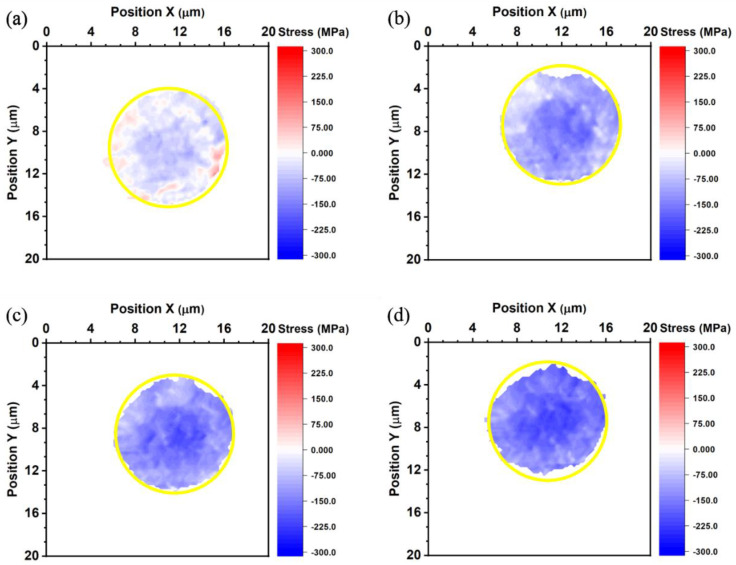
Thermal stress maps of the top die measured at (**a**) 27, (**b**) 100, (**c**) 150, and (**d**) 200 °C. The yellow circles represent the site of the bump.

**Figure 8:**
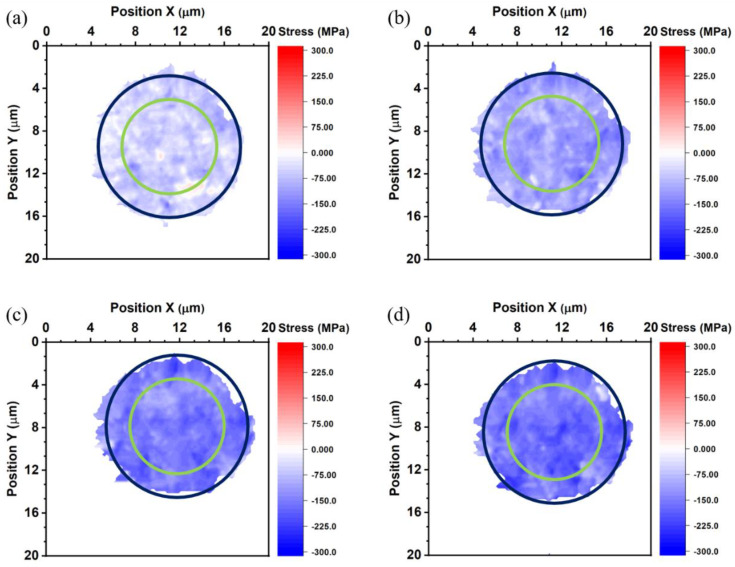
Thermal stress maps of the bottom die measured at (**a**) 27, (**b**) 100, (**c**) 150, and (**d**) 200 °C. The blue and green circles represent the sites of the bump and the RDL, respectively.

**Figure 9:**
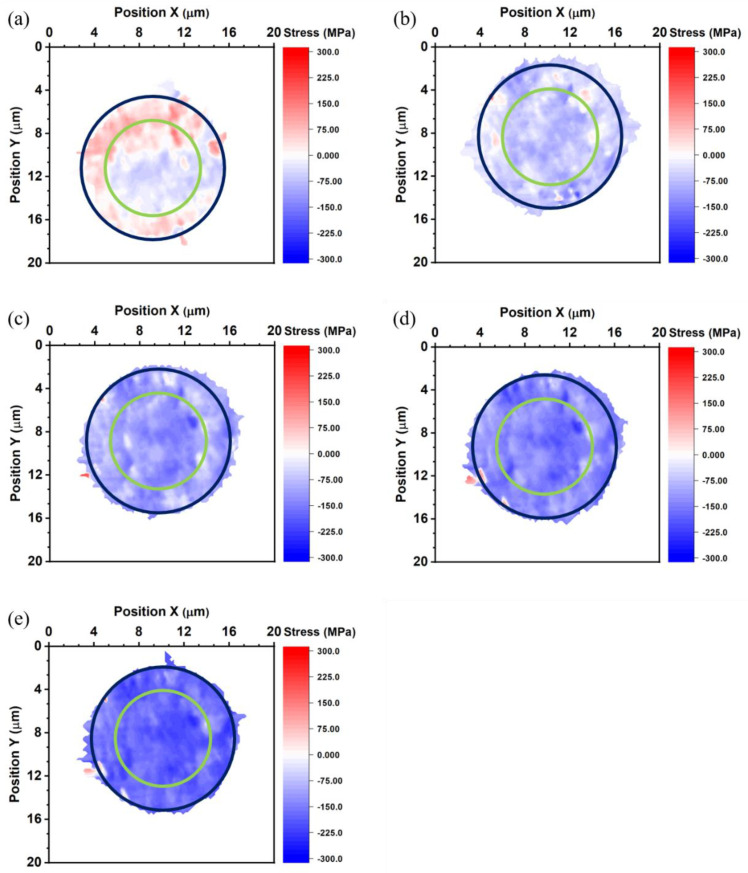
Thermal stress maps of the bonded joint measured at (**a**) −55, (**b**) 27, (**c**) 100, (**d**) 150, and (**e**) 200 °C. The blue and green circles represent the sites of the bump and the RDL, respectively.

(3) σ=E×ε

where *σ* is thermal stress, *E* is Young’s modulus, and ε is thermal strain. The thermal stress of the top die Cu pad, bottom die Cu pad, and bonded Cu joint is shown in Figure 7, Figure 8 and Figure 9. The average thermal stress of the top die Cu pad, bottom die Cu pad, and the bonded Cu joint is listed in Table 2. The average thermal stress of the top die Cu pad is −25.2, −98.0, −124.6, and −155.4 MPa measured at 27, 100, 150, and 200 °C, respectively. The average thermal stress of the bottom die Cu pad is −51.8, −103.6, −147.0, and −158.2 MPa measured at 27, 100, 150, and 200 °C, respectively. Additionally, the average thermal stress of the bonded Cu joint is 9.8, −54.6, −100.8, −131.6, and −169.1 MPa measured at −55, 27, 100, 150, and 200 °C, respectively. In the Cu/SiO_2_ structure, the Cu pads were confined by the surrounding SiO_2_, so the thermal expansion behavior of the Cu pads would be inhibited. It is noteworthy to state that the stress values we measured were in the vertical direction of the Cu bumps because we adopted the diffraction spots from the (222) planes, as shown in Figure 2.

One might expect that the stress in Cu bumps in the top and bottom dies would be small in the vertical direction because the Cu may expand in the direction of the top free surface and release the stress imposed by the surrounding SiO_2_ layer. However, the stress values we measured are over 100 MPa in compression at temperatures higher than 100 °C. This is explained as follows. As the temperature increases, the Cu should expand more than the surrounding SiO_2_ because the CTE of Cu is much larger than that of the SiO_2_. As shown in Figure 3b,c, the Cu bumps would experience compressive stress from the lateral surrounding SiO_2_ layer. The Cu might relieve the stress through the expansion to the top surface. However, the Cu bumps adhere to the sidewalls of the SiO_2_ quite well. Thus, the vertical expansion might be limited to some extent. Therefore, the Cu bumps were under high compressive stress in the vertical direction.

**Figure 10:**
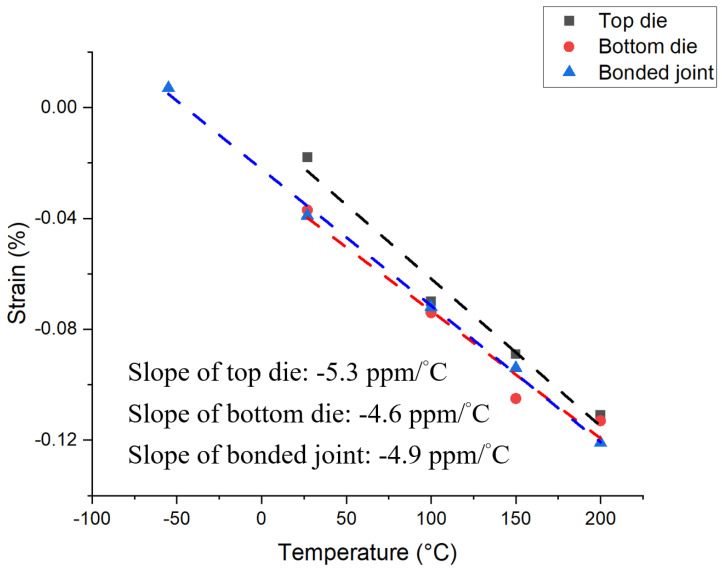
The average thermal strain of the top die, bottom die, and bonded joint.

With the strain values at various temperatures, one can calculate the effective CTE of the Cu bumps embedded in the SiO_2_ layer. Figure 10 plots the average thermal strain of the top die, bottom die, and the bonded joint against temperature. Since the strains we calculated were using Equations (1) and (2), the slopes of the fitting lines in Figure 10 represent the mismatch of the CTE between the confined Cu bumps and free-standing Cu. The slope of the top die Cu pad, bottom die Cu pad, and bonded Cu joint is −5.3, −4.6, and −4.9 ppm/°C, respectively. Then, one can obtain the effective CTE of the Cu pad by adding the above value to the CTE of free-standing Cu, which is 16.99 ppm/°C. Therefore, the effective CTE of the top die Cu pad, bottom die Cu pad, and the bonded Cu joint is calculated to be 11.7, 12.4, and 12.1 ppm/°C, respectively. In our previous study, we found the effective CTE of the Cu line is about 21 ppm/°C, which is greater than the CTE of the free-standing Cu [46]. Because the Cu line is not embedded in the SiO_2_, the effective CTE is higher. Moreover, we can use the effective CTE to calculate the expansive height due to thermal expansion at the bonding temperature with Equation (4).

(4) $$\Delta L=\alpha_{eff}L_{0}∆T$$

where Δ*L* is the difference in height, *α_eff_* is the effective CTE, *L*_0_ is the thickness of Cu pad, and ∆*T* is the temperature difference. We substituted *α_eff_* as 11.7 and 12.4 ppm/°C for the top die Cu pad and the bottom die Cu pad, *L*_0_ as 1250 nm, and ∆*T* as 173 °C. Then, we obtained the expansive height of the top die Cu pad and the bottom die Cu pad as 2.5 and 2.7 nm, respectively. These values represent the maximum recess of the top die and the bottom die Cu pad, which cannot be over 2.5 and 2.7 nm for good bonding quality. In addition, as the pitch and the thickness of the Cu pad shrink, the maximum recess should decrease as well. As the size of Cu pad decreases to 500 nm, the calculated thermal expansion should be less than 4 nm [47]. However, the constraint of the surrounding SiO_2_ would be aggravated in smaller bumps. Thus, the effective CTE and the behavior of thermal expansion in fine pitch needs more investigation in the future.

The thermal stress results provide a fundamental understanding of the mechanism of the Cu–Cu bonding. For previous study on the Cu–Cu bonding using blanket films, an external pressure ranging from 1 MPa to tens of MPa was applied to the Cu films at elevated temperatures, which is called “thermal compression bonding”, and bonding was achieved after approximately 1 h of the creep process. However, for real applications in microelectronic devices, the Cu bumps are embedded in dielectric films, as illustrated in Figure 1, so a hybrid bonding is needed. Although there is no external pressure applied to the top and the bottom wafer at the bonding temperature at 200 °C in this study, the local stress in the Cu bumps is as high as −169.1 MPa, which is generated by the mismatch of CTE in the heterogeneous integrated structure. The high thermal stress provides the pressure needed for the Cu–Cu diffusion bonding. On the other hand, the local high pressure may cause failure on the fragile, porous, low-K dielectric materials underneath the Cu joints. Therefore, managing the pressure during Cu–Cu bonding is an essential task for the microelectronic industry.

## 4. Conclusions

We used X-ray nano-diffraction to measure the thermal stress in a (111)-oriented NT-Cu bump of the hybrid Cu/SiO_2_ joint at various temperatures. At room temperature, the average thermal strain is compressive, and as the temperature increases, the thermal compressive strain increases. The average thermal stress of the top die Cu pad measured at 27, 100, 150, and 200 °C was −51.8, −103.6, −147.0, and −158.2 MPa, respectively. The average thermal stress of the bottom die Cu pad measured at 27, 100, 150, and 200 °C was −25.2, −98.0, −124.6, and −155.4 MPa, respectively. The average thermal stress of the bonded Cu joint measured at −55, 27, 100, 150, and 200 °C was 9.6, −54.1, −101.3, −131.3, and −169.1 MPa, respectively. In addition, from the slope of the average thermal strain of Cu pad against temperature, one can obtain the effective CTE of Cu bumps confined in the SiO_2_. The measured effective CTE of the top die Cu pad, bottom die Cu pad, and bonded Cu joint was 11.7, 12.4, and 12.1 ppm/°C, respectively, which is much lower than the literature value of 16.99 ppm/°C for the free-standing Cu. The high thermal stress at 200 °C provides the driving force for the Cu–Cu diffusion bonding. These results provided a new insight of the effect of thermal stress on Cu/SiO_2_ hybrid bonding.

## Figures and Tables

**Table 1:** Summary for the average thermal strain of Cu via in the top die, bottom die, and bonded joint at various temperatures.

Unit: %	*T*/°C −55	27	100	150	200
Top die	N/A	−0.018	−0.07	−0.089	−0.111
Bottom die	N/A	−0.037	−0.074	−0.105	−0.113
Bonded joint	0.007	−0.039	−0.072	−0.094	−0.121

**Table 2:** Summary for the average thermal stress in Cu via in the top die, bottom die, and bonded joint at various temperature.

Unit: MPa	*T*/°C −55	27	100	150	200
Top die	N/A	−25.2	−98.0	−124.6	−155.4
Bottom die	N/A	−51.8	−103.6	−147.0	−158.2
Bonded joint	9.8	−54.6	−100.8	−131.6	−169.1

## Data Availability

The authors declare that they have no known competing financial interests or personal relationships that could appear to influence the work reported in this paper.
